# Peer support and shared decision making in Open Dialogue: Opportunities and recommendations

**DOI:** 10.3389/fpsyg.2022.1059412

**Published:** 2022-12-08

**Authors:** Marta Chmielowska, Nell Mannocci, Alexander Tansel, Yaara Zisman-Ilani

**Affiliations:** ^1^Centre for Outcomes Research and Effectiveness, Research Department of Clinical, Educational and Health Psychology, University College London, London, United Kingdom; ^2^North East London NHS Foundation Trust Research and Development Department, London, United Kingdom; ^3^Division of Psychology and Language Sciences, Department of Clinical, Educational and Health Psychology, University College London, London, United Kingdom; ^4^Social and Behavioral Sciences, Temple University College of Public Health, Philadelphia, PA, United States

**Keywords:** peer support, shared decision making, Open Dialogue, mental health, mental illness, peer support workers, lived experience

## Abstract

Open dialogue (OD) is a person-centred social network model of crisis and continuing mental healthcare, which promotes agency and long-term recovery in mental illness. Peer support workers who have lived experience of mental illness play a key role in OD in the UK, as they enhance shared understanding of mental health crisis as part of the OD model and provide a sense of belonging and social inclusion. These elements are in alignment with the shared decision making (SDM) approach in mental health, which focuses on person-centred communication in treatment decision-making. The previously documented benefits of peer-led SDM include increased engagement with services, symptom reduction, increased employment opportunities, and reduced utilization of mental and general health services. While the contribution of peer support and SDM principles to OD has been acknowledged, there is only a small body of literature surrounding this development, and little guidance on how peer support can enhance treatment decision-making and other aspects of OD. This viewpoint, which was co-authored by people with lived experience of mental illness, clinicians, and researchers, discusses practical implications and recommendations for research and training for the provision of a co-produced OD model grounded in peer support and SDM.

## Introduction

Open dialogue (OD) is a person-centred model of mental health care that is based on collaboration between a clinician, a person experiencing a mental health crisis and their social network (SN; e.g., family members, friends, and carers) (Seikkula et al., 2001; Seikkula and Olson, 2003; Olson et al., 2014; Pilling et al., 2022). OD is both a therapeutic practice and a way of organizing services (Seikkula et al., 2003). The central means of intervention delivery are through network meetings: reflective conversations between people who access mental health services and their social or professional environments to enable a mutual and deeper understanding of the current crisis, as well as to draw on the resources of the network and facilitate inherently democratic and transparent decision-making (Aaltonen et al., 2011; von Peter et al., 2021). OD aims to protect and promote the autonomy of people who access mental health services: respect their choices, priorities, and values (World Health Organization, 2021).

Peer support is increasingly recognized as an important and transformative element of mental health care (Maruthappu et al., 2014) provided by people with lived experience of mental illness, which involves giving and receiving help based on self-determination, respect and social inclusion, shared responsibility and mutual agreement on what could be helpful (Mead et al., 2001; Dennis, 2003). Peer support exists along a continuum, from informal, mutual relationships of connection and support at one end to more formal relationships in which people with lived experience of mental illness are employed to help at the other (Bradstreet, 2006; Davidson et al., 2006). The lived experience of peer support workers (PSWs) can improve decision-making by mirroring people who access mental health services to voice their concerns and priorities, values and preferences (Cleary et al., 2018).

In the UK, peer support makes a unique contribution to OD as PSWs are trained to take on dual roles of experts by experience and as community navigators within their clinical teams (Razzaque and Stockmann, 2016; Bellingham et al., 2018). As experts by experience, PSWs have a psychotherapeutic role alongside clinicians in network meetings where they engage in self-reflections to help people who access mental health services and their SNs feel heard, respected and validated. PSWs can therefore facilitate transparent decision-making about treatment and recovery through open dialogue and collaboration between all members of a SN meeting. Secondly, as community navigators, they have a more professional role, in which their expertise is used to help individuals with a limited SN link up with people who currently receive (or have received) support from local mental health services. It will be a self-help community that the PSWs facilitate and bring forward as a resource for those who can benefit from it.

The dual role of PSWs in OD is in alignment with the shared decision making (SDM) approach in mental health, which focuses on person-centred communication in treatment decision-making, with the goal of improving experience of care as well as clinical and functional outcomes (Zisman-Ilani et al., 2021c; Zisman-Ilani and Byrne, 2022). Indeed, the key principles of SDM in OD include (1) the reduction of power asymmetries between a clinician and a person accessing mental health services; (2) the recognition that there are at least two expert participants: a person with lived experience, a clinician with professional expertise and a SN member; (3) the expression of preferences of the person accessing mental health services for involvement in decision-making and the expression of their specific values that could guide the decision; (4) the discussion of at least two treatment options; (5) making or postponing a decision that is consistent with the patient’s goals, preferences and values; and (6) accepting that the patient’s choice of treatment plan may differ from the clinician’s recommendation (Zisman-Ilani et al., 2021c).

However, bringing together peer support and OD may not necessarily be straightforward in practice. Barriers to the successful implementation of peer support in OD may include lack of role clarity (Crane et al., 2016), prioritization of clinical decision-making (including prescribing decisions), (Zisman-Ilani et al., 2017) stigma and negative attitudes of clinicians, (Wheeler et al., 2020) lack of clear boundaries between PSWs and people who access mental health services, (Miyamoto and Sono, 2012) poor team functioning, limited career opportunities, and inadequate training, supervision, and logistical support for PSWs (Vandewalle et al., 2016). Therefore, the development of a co-produced OD model grounded in peer support and SDM can help overcome these barriers and embed a culture-change in mental health services.

We, the authors of this viewpoint, have an interest and experience in receiving and delivering OD treatment. We believe that potential contributions of peer support and SDM to OD include the development of meaningful relationships that empower people who access mental health services and their SN to manage their own care and treatment (Bellingham et al., 2018); the promotion of democratic partnerships between clinicians and people who access mental health services, and the reduction of clinical hierarchies in mental health services (Razzaque and Stockmann, 2016); the humanization of mental health services where delivering person-centred care is a top priority, (Youngson and Blennerhassett, 2016) the promotion of greater understanding of peer support perspectives, (Stockmann et al., 2019) and the promotion of recovery-oriented care (Razzaque and Stockmann, 2016; Bellingham et al., 2018). Therefore, the pairing of the two approaches and their implementation and adoption in the UK mental health services has a revolutionary potential to change the way we respond to human distress.

### Peer support and shared decision making build meaningful and empowering relationships in mental health services

The development of meaningful relationships is central to peer support in OD so that people who access mental health services can feel supported to reflect and express their preferences and views during the decision-making process (Adame and Leitner, 2008). PSWs bring together both social and professional networks by establishing connections between clinicians, people who access mental health services and their family. The role of a clinician focuses more on maintaining established relationships with members of the SN (Razzaque and Stockmann, 2016) than on rushing to agree or provide expert advice. There is also a strong emphasis on the mobilization of resources within people who access mental health services and their SN to increase feelings of agency and the ability to develop and maintain mutually supportive relationships in the longer term (Pilling et al., 2022). Consequently, people who access mental health services and their SN are encouraged to make their own decisions about their health and treatment, demonstrating the emancipatory and empowering potential of peer support in OD.

### Peer support and shared decision making promote democratic partnerships in mental health services

Peer support in OD engages in dialogue from different perspectives and tries to privilege all voices, which would necessarily include the voices of clinicians, people who access mental health services, members of their SN and PSWs (Bellingham et al., 2018). Peer support in OD also asks clinicians to abandon the position of expert-by-knowledge and practice from a place of “not knowing” (Anderson, 1990). By not having prior medical education and training and yet finding the courage to speak out and share their views and experiences of mental illness, PSWs can help clinicians give up the authoritarian role, lean into uncertainty, tolerate risks, (Scott, 2011) and facilitate spaces to discuss treatment openly and democratically. PSWs can promote democratic partnerships, especially in more complex decision-making situations, such as psychiatric medication management, as SDM is often perceived as a risk to clinicians due to liability and clinical errors (Zisman-Ilani et al., 2021b). Indeed, research into how SDM occurs at psychiatric medication management meetings has shown that clinicians often use persuasion in encounters with people who access mental health services, and concerns about adverse effects are often ignored (Quirk et al., 2012; Kaminskiy and Finlay, 2019). Peer-led SDM in OD can therefore place a greater emphasis on personal meanings and a broader psychological and social understanding of medication, strengthening the ideal of a meeting of different experts (i.e., experts-by-experience versus experts-by-knowledge) and the value of experiential knowledge encounters (Ramon et al., 2017; Leendertse et al., 2021).

### Peer support and shared decision making humanize mental health services

Peer support in OD embodies the key principles of person-centred care, such as dignity, compassion, respect, choice, and empowerment. Peer support in OD emphasizes co-production and active citizenship in recovery (Ramon, 2018) to promote a better understanding of the perspectives of lived experience of mental illness (Stockmann et al., 2019). PSWs offer people who access mental health services the opportunity to share common experiences of stigma and discrimination, to help them develop new insights into their own mental health and protect them from feelings of shame, social alienation and isolation (Bellingham et al., 2018). PSWs ask clinicians to reflect on their own lived experience of mental illness whenever possible and bring more of themselves into network meetings (Olson et al., 2014; Stockmann et al., 2019). PSWs do not share the systemic culturalization of clinicians and have a more nuanced understanding of mental illness that can inform care practices. PSWs view crisis as temporal and episodic, and recovery as a deeply social, unique, and shared process (Baumgardt and Weinmann, 2022). PSWs can therefore restore human values by focusing on listening and responding to the whole person in a context rather than primarily focusing on their symptoms.

### Peer support and shared decision making are the key components of recovery-oriented care

Peer support in OD shares common values of the recovery model of mental illness such as hope, self-determination, empowerment, community integration and advocacy (Onken et al., 2002). These values challenge personal narratives of distress by exposing the need for recovery from iatrogenic harm and restrictive treatments (Bellingham et al., 2018). By sharing their experiential knowledge, PSWs support people who access mental health services in initiating and maintaining recovery and improving the quality of their personal, family and social lives (White, 2009). Since OD is intended to reflect the core interests of people who access mental health services, questions arise about how the outcomes used to evaluate peer support and SDM in OD align with the outcomes they value. The lack of a clear definition of peer support and SDM in OD, (Shalaby and Agyapong, 2020; Zisman-Ilani and Byrne, 2022) the holistic nature of recovery outcomes, and the fact that mental illness affects almost all aspects of life (e.g., housing, SNs, employment, education, mental health, and health care treatment) have led to different conclusions on which areas should receive the most attention and why (Whitley and Drake, 2010).

## Discussion

Peer support and SDM are increasingly recognized as the central pillars of recovery from mental illness. This is based on the important premise that the meaning of recovery can be different for everyone, and that people can benefit from sharing experiences, being listened to and respected, being supported to find meaning in their experiences and a path to recovery that works for them, ultimately enabling them to lead a fulfilling and satisfying life (World Health Organization, 2021). Therefore, recent efforts to include peer support and SDM in OD hold promise and highlight different points of convergence between them. Both OD and peer support practices are concerned with different meanings of distress, emphasize collaboration and democracy, and SDM in care and treatment for mental illness. Furthermore, the OD principle of “tolerating uncertainty” is not entirely different from the principles of peer support of “not knowing” and “dignity of risk,” which support self-determination and seek to avoid risk-averse practices (Mead and Hilton, 2003; Repper and Carter, 2011; Scott et al., 2011). Nevertheless, research attempts to determine how peer support can enhance SDM and other aspects of OD highlight important challenges and opportunities that researchers and health care providers are encouraged to consider.

### The core principles of peer support and shared decision making in Open Dialogue

Peer support workers are employed members of the clinical team who make a unique contribution to network meetings by using their lived experience to engage people who access mental health services in their treatment. Nevertheless, the current descriptions of PSW roles are too general, and there is little rationale for positioning peer support in the OD approach broadly and in network meetings, more specifically. The study of the impact of peer support on mental health using statistical approaches is therefore limited, and does not fully take into account people and their unique characteristics, as it mainly emphasizes the importance of qualitative research in this field (Bellingham et al., 2018). Clarifying the core values and principles of the PSW role in OD will ensure that, as peer support grows, it grows with integrity to its founding values and remains distinct from other mental health interventions that are not based primarily on the person’s own life experiences.

### The core outcome set for Open Dialogue research and clinical practice

Clinical outcomes such as psychiatric hospitalizations or psychiatric symptoms remain a focus of peer support and SDM in mental health research, and contribute to a mixed evidence base for the effectiveness of OD interventions for the treatment of mental illness, with recovery-oriented outcomes such as empowerment, self-efficacy, and hopefulness being the main outcomes (Salyers and Zisman-Ilani, 2020; Zisman-Ilani et al., 2021a). The lack of validated outcome measures uniquely developed to assess peer support and SDM in mental health is a critical factor that contributes to the limited use of recovery-oriented peer support and SDM outcomes. A useful strategy is to consider which outcomes are valued by the people who use services, and to develop an evaluation approach based on these objectives. Person-driven measurement approaches and more participatory research methods can improve both the quality and impact of health and mental health services. Therefore, an agreement must be reached on a core outcome set for measuring peer support and SDM as part of recovery-oriented care in OD (Wheeler et al., 2020).

## Conclusion

This viewpoint emphasizes the potential contributions of peer support and SDM to the provision of a co-produced OD model. Peer support and SDM are at the heart of person-centred care and personal recovery. An updated OD model grounded in peer support and SDM sets a new direction for OD research, with the emphasis on developing and validating peer support and SDM measures with and for people who access mental health services. Such a model goes beyond simply pairing the two approaches and deliberately requires the inclusion of a competence framework that considers the strengths of peer support and OD. This new framework can provide a better understanding of how PSWs add value to the competences of OD teams and services. It can also protect people working in PSW roles from being asked to work in inappropriate ways, either beyond their competence or in a way that does not make the best use of their skills.

## Data availability statement

The original contributions presented in this study are included in the article/supplementary material, further inquiries can be directed to the corresponding author.

## Author contributions

MC wrote the first draft of the manuscript. YZ-I read and revised the draft further. NM and AT served as a driving force behind the concept and provided guidance on how to structure the manuscript. All authors contributed to the article and approved the submitted version.
